# Fibromuscular dysplasia of the brachial artery diagnosis by Doppler ultrasound: a case report

**DOI:** 10.1590/1677-5449.202500142

**Published:** 2026-01-12

**Authors:** Mariana Jordão França, Luciana Akemi Takahashi, Graciliano José França

**Affiliations:** 1 Universidade Positivo – UP, Curitiba, PR, Brasil.; 2 Universidade Federal do Paraná – UFPR, Curitiba, PR, Brasil.; 3 Pontifícia Universidade Católica do Paraná – PUCPR, Curitiba, PR, Brasil.

**Keywords:** fibromuscular dysplasia, brachial artery, doppler ultrasound

## Abstract

Fibromuscular dysplasia (FMD) is a disease of non-inflammatory vascular origin that occurs in medium-sized arteries. Its cause is still unknown. The disease mostly affects middle-aged Caucasian women and, in most cases, involves the renal artery. Disease involvement in the arteries of the upper and lower limbs is rare. We report a case of fibromuscular dysplasia of the medial layer of the brachial artery in an asymptomatic patient using Doppler Ultrasound.

## INTRODUCTION

Fibromuscular dysplasia (FD) was first described by Leadbetter and Burkland, in 1938, as a non-inflammatory and non-atherosclerotic vascular disease characterized by narrowing of medium-caliber arteries secondary to fibrodysplastic changes involving the arterial layers.^1^ It is classified on the basis of which layer is primarily involved: the intima, media, or adventitial. Tunica media injuries occur in 90% of cases and are associated with slow progression. Involvement of this tunica is recognized by the classic “string of beads” appearance, in which there is alternate thickening and narrowing of the arterial segments with FD involvement. Involvement of the tunica intima manifests with focal and concentric stenosis, while adventitial involvement appears as tubular stenosis. The disease can also provoke rupture of the internal elastic lamina, associated with presence of an aneurysm, or can lead to dysplastic injuries, resulting in stenosis.^2^ Presence of at least one focal or multifocal arterial injury is necessary for a diagnosis of FD. Isolated presence of aneurysm, dissection, or tortuosity is insufficient to make a diagnosis.^3^

Fibromuscular dysplasia predominantly affects Caucasian lean women aged from 15 to 50 years.^2^ Approximately 80-90% of cases are in women^3^ and etiology remains unknown. The arteries most commonly affected by FD are the renal and the carotid.^4^ It is estimated that the prevalence of this condition is lower than 1% of the population and 60-75% of cases involve the renal arteries. In addition, cases have also been described with involvement of the visceral, iliac, subclavian, popliteal, and brachial arteries.^2^ Involvement of the arteries of the upper limbs is considered rare in FD.^4^ The gold standard for imaging investigation is catheter angiography,^5^ although other methods can also be used, such as computed angiotomography, magnetic resonance angiography, and Doppler ultrasound.^2^ We describe the case of a female patient with asymptomatic unilateral FD of the brachial artery, diagnosed using Doppler ultrasound.

The study was approved by the Ethics Committee at our institution (opinion number 6.860.303). A free and informed consent form was signed covering all procedures involving human beings. The patient also signed a consent form specifically covering publication of ultrasound images and the case report.

## CASE DESCRIPTION

The patient was a 64-year-old female with a history of headaches associated with suspected temporal arteritis, which had been treated with prednisone 60 mg/day, resulting in improvement of the symptoms. However, no biopsy of the temporal artery had been performed to confirm the diagnosis. On physical examination, the patient’s distal pulses were weak in the right upper limb. The Rheumatology service referred her to the vascular surgery team for arterial Doppler ultrasound of the upper limb, to investigate the weak pulses. She denied having claudication of the upper limbs.

**Figure 1:**
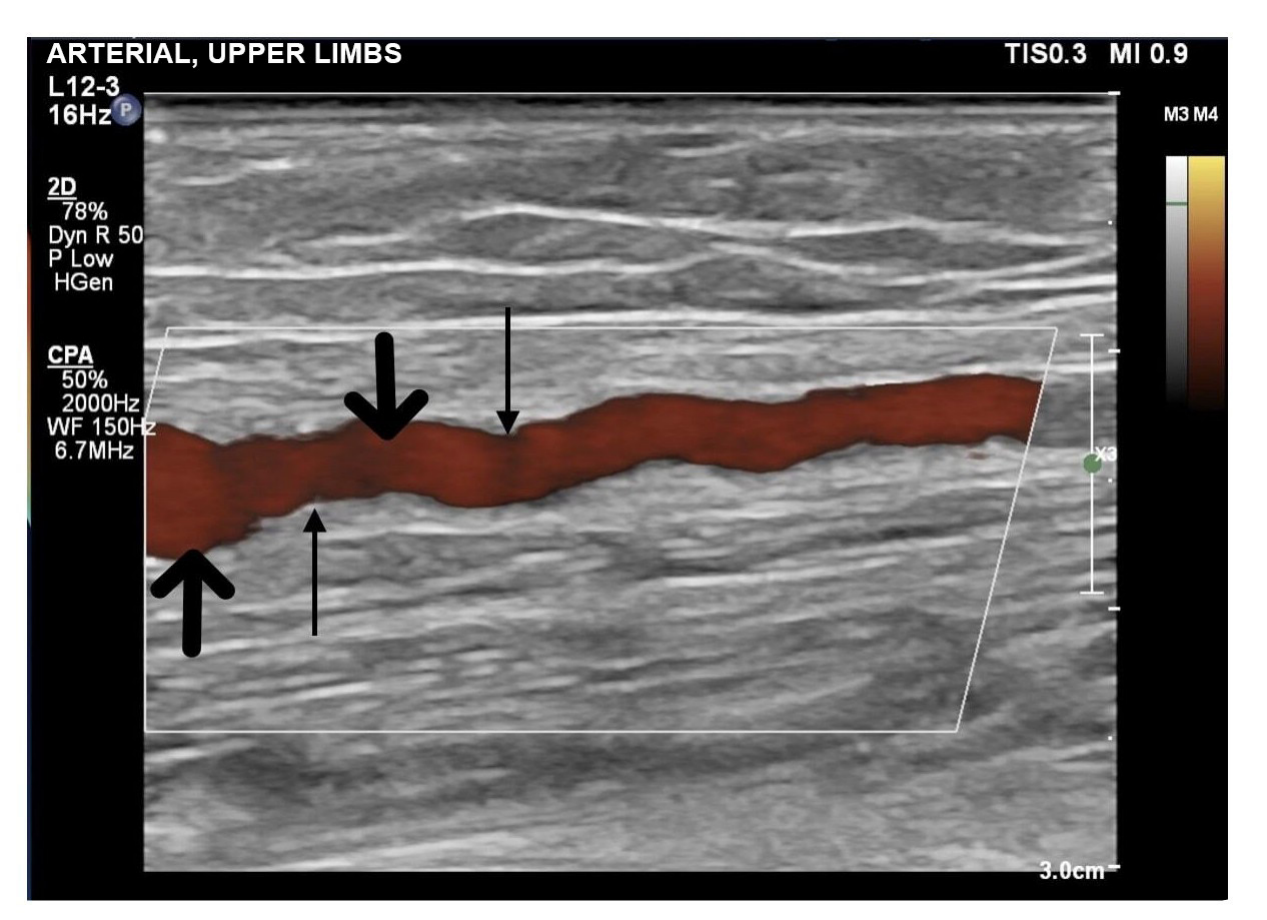
Power Doppler image of the right brachial artery demonstrating small areas of stenosis interspersed with dilations, in the “string of beads” pattern. The thicker arrows indicate dilated areas and the thinner arrows indicate areas of stenosis.

**Figure 2:**
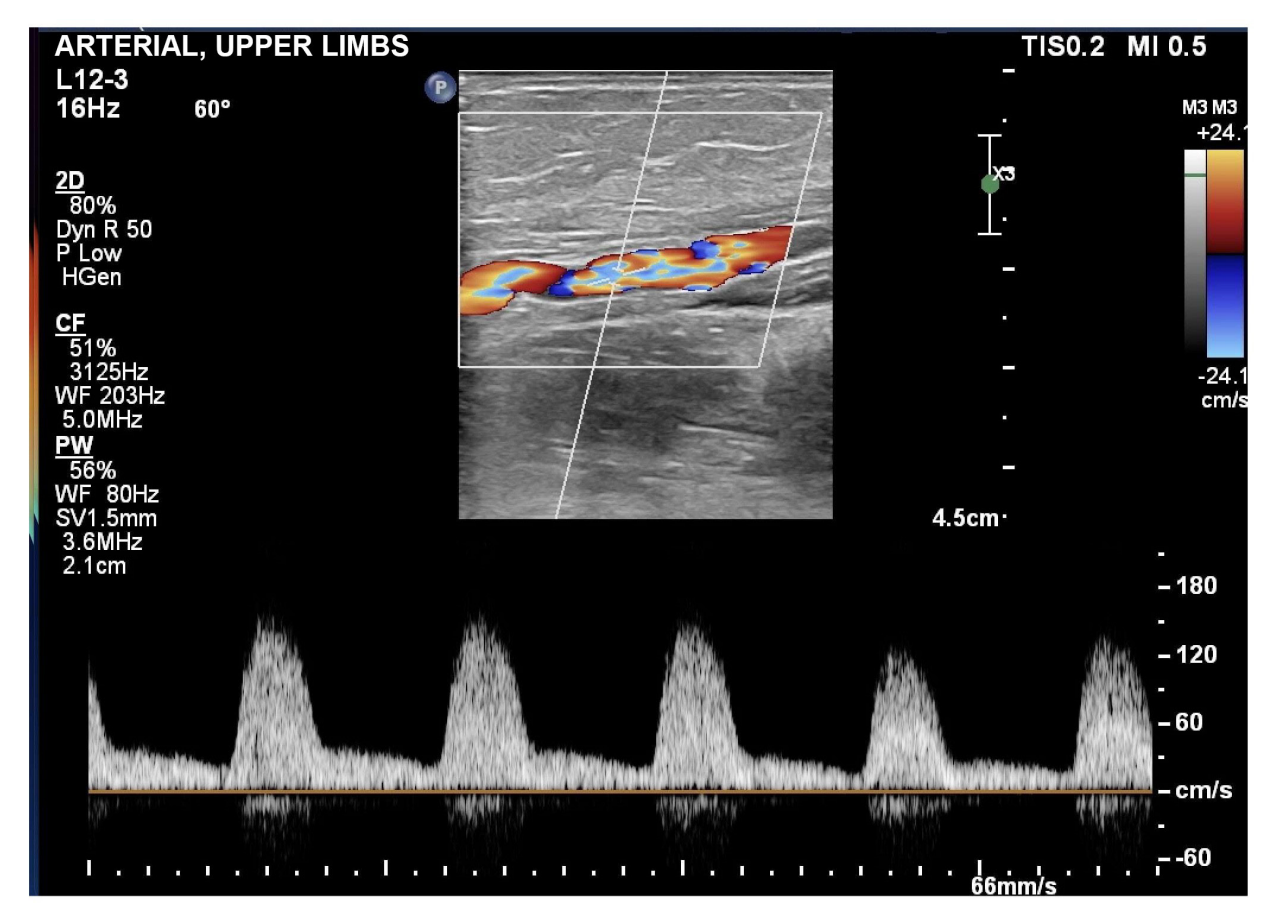
Brachial artery with areas of aliasing in color mode, increased peak systolic velocity, and reduced arterial resistance index, compatible with hemodynamically significant and sequential stenoses.

**Figure 3:**
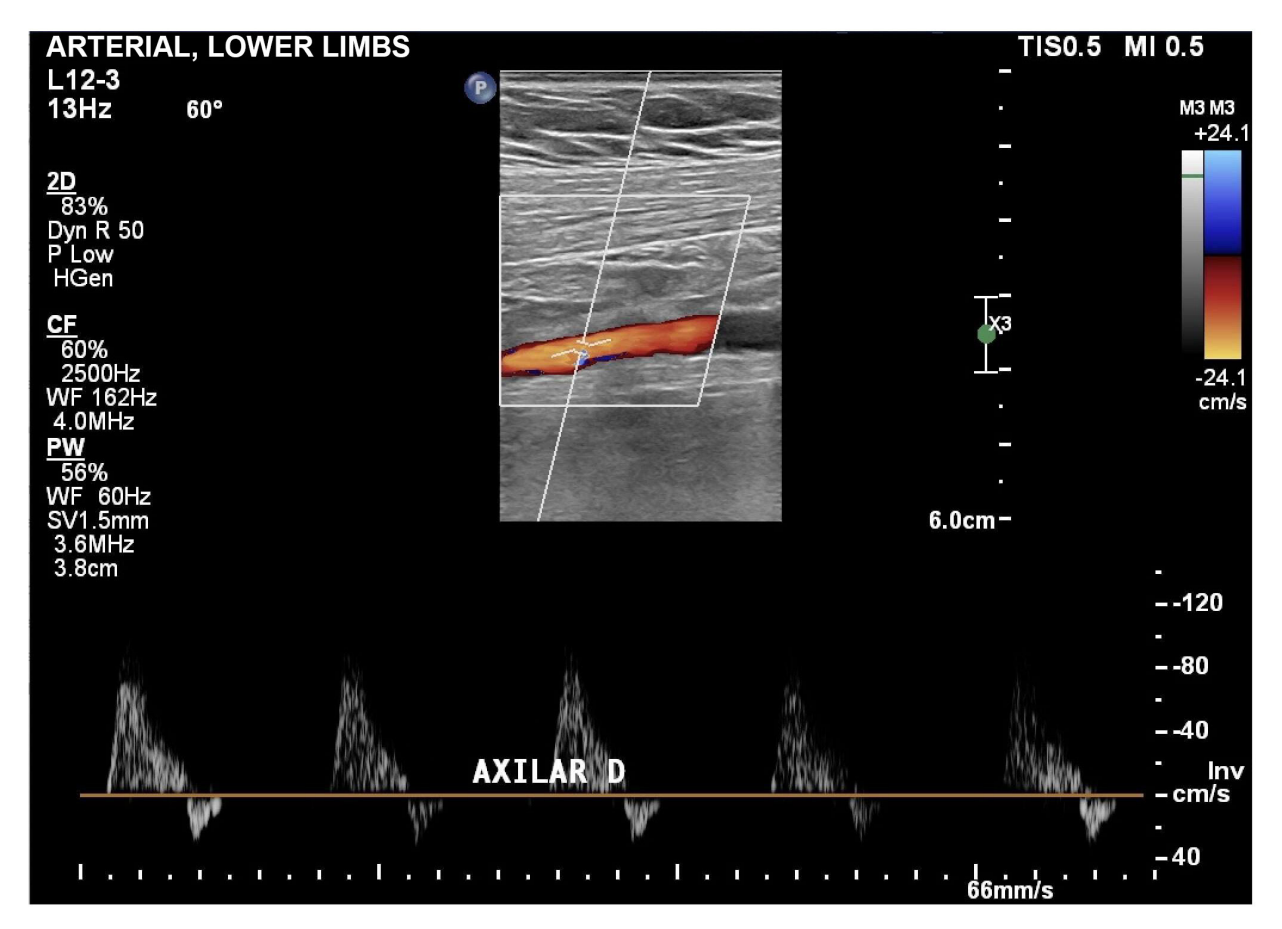
Doppler ultrasound of the right axillary artery, showing curves de with normal morphology and no signs of stenosis.

**Figure 4:**
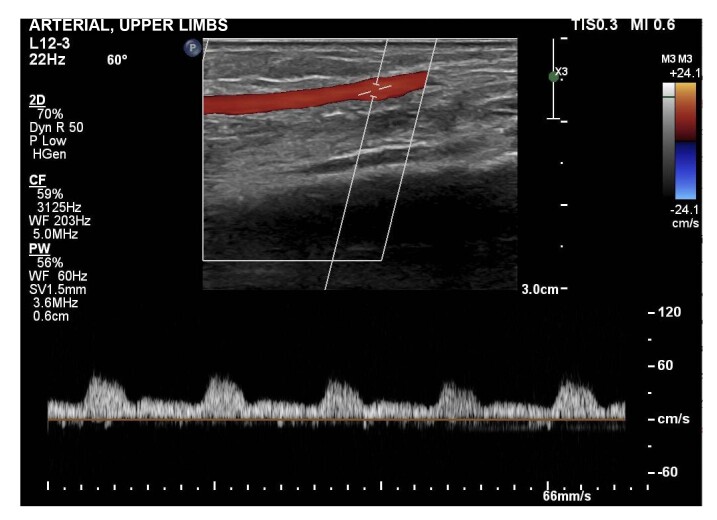
Doppler ultrasound of the right radial artery, showing signs of hypoflow caused by significant proximal stenosis.

The arterial Doppler ultrasound showed multiple small dilatations of the right brachial artery, without presence of atheromas (Figure 1), interspersed with hemodynamically significant stenosis, causing aliasing in color mode and increased peak systolic velocity seen in Doppler mode (Figure 2). These findings are suggestive of the “string of beads” pattern and compatible with a diagnosis of FD. The subclavian and axillary arteries of the right upper limb exhibited normal wave morphology (Figure 3). The radial and ulnar arteries in the same limb did not show local stenosis; however, Doppler flow measurement revealed signs of hypoflow, confirming the diagnosis of hemodynamically significant stenosis of the brachial artery (Figure 4). There was no evidence of FD in the left brachial artery. The left subclavian, axillary, brachial, radial, and ulnar arteries were all patent, with straight walls, no atheromas, and flow within normal limits, with no sign of stenosis. No additional Doppler ultrasound examinations were performed to assess the carotid arteries. The patient remains asymptomatic and in clinical follow-up.

## DISCUSSION

The etiology of FD remains unknown, although it is believed that there is a genetic component, based on the predominance of Caucasian women affected, which may be related to the HLA-DRw6 histocompatibility antigen.^2^ Suzuki et al. reported a familial case of bilateral FD of the brachial artery involving a mother and daughter, which adds weight to the genetic factor hypothesis. The same authors also mention studies suggesting autosomal dominant inheritance and reduced penetration in males.^6^ Additional studies are needed to improve understanding of the role played by genetic variations in FD pathogenesis. There is not currently a specific genetic test for the disease and there are no recommendations for genetic testing of asymptomatic relatives of patients with FD.^3^ The predominance among women and discovery of some cases during pregnancy also suggests that estrogen may play a role in the pathogenesis of this vascular disease.^7^

Other cases have been associated with smoking, coagulation disorders, factor V Leiden mutation, presence of antiphospholipid antibodies, and mechanical stress.^8^ Additionally, physical stress and episodes of trauma may also contribute to development of FD, since these injuries provoke ischemia in the artery wall and compromise the vasa vasorum.^9^

Diagnosis of the disease in upper extremities may be incidental in asymptomatic patients, or may occur after clinical suspicion has been aroused. In this segment, FD primarily involves the brachial artery, although cases have been reported in which the axillary, subclavian, radial, and ulnar arteries were involved. The most common pattern of involvement is multifocal, with a majority of asymptomatic patients, and presentation is frequently bilateral.^3^ Manifestations of FD in the upper extremities include cyanosis, Raynaud phenomenon, paresthesia, weakness, presence of pulsating mass, ulcer, or, in more severe cases, distal gangrene of the fingers associated with microembolism.^4^ These symptoms may be caused by changes to arterial flow or because of compression of nerve structures.^1^ Physical examination may identify attenuated peripheral pulses, differences in blood pressure between upper limbs, and presence of arterial murmur.^3^ It is important to differentiate between FD and atherosclerosis, since FD may present symptoms similar to those of atherosclerosis in patients with intermittent claudication.^2^

These patients must be followed-up regularly, because of the progressive character of FD. Treatment is only recommended for symptomatic individuals.^2^ A prospective study conducted by Nguyen et al. with 22 women with FD of the upper extremity reported that 51.7% of them were asymptomatic.^4^

There are a number of interventions possible for symptomatic patients, ranging from drug-based treatments to invasive procedures. Drugs used include platelet antiaggregants, anticoagulants, and calcium channel blockers.^4^ The first international consensus on diagnosis and management of FD recommends that patients with FD who do not have contraindications to antiplatelet treatment should be given aspirin at a daily dose of 75-100 mg with the objective of preventing thrombotic and thromboembolic complications.^3^ Invasive procedures indicated for symptomatic patients include transluminal percutaneous angioplasty, catheter-directed thrombolysis, sympathectomy, and surgical bypass.^4^

Since diagnosis and management of FD in the upper limbs are primarily founded on case reports or case series with few participants, there is no consensus on treatment.^4^ Endovascular interventions tend to be preferred for short segments, while open surgery is indicated for longer segments.^10^

The prospective study by Nguyen et al. concluded that drug-based treatment alone has limited efficacy for complete relief of symptoms. In these cases, the initial invasive intervention may be primary angioplasty. In more serious cases, more than one intervention may be necessary, opting for a surgical bypass, or, in some cases, sympathectomy.^4^

Doppler ultrasound is a rapid, low-cost, and widely available assessment method that constitutes one possible imaging exam for investigating FD. Moreover, it can differentiate FD from atherosclerosis and show whether involvement is bilateral or unilateral.

## Data Availability

Todos os dados gerados ou analisados e imagens de ultrassom Dopple estão incluídos neste artigo e/ou no material suplementar.

## References

[1] De Waele M, Lauwers P, Hendriks J, Van Schil P (2012). Fibromuscular dysplasia of the brachial artery associated with unilateral clubbing. Interact Cardiovasc Thorac Surg.

[2] de Carvalho Pontes T, Rufino GP, Gurgel MG, de Medeiros AC, Freire EA (2011). Displasia fibromuscular: um diagnóstico diferencial para as vasculites. Rev Bras Reumatol.

[3] Gornik HL, Persu A, Adlam D (2019). First International Consensus on the diagnosis and management of fibromuscular dysplasia. Vasc Med.

[4] Nguyen N, Sharma A, West JK (2017). Presentation, clinical features, and results of intervention in upper extremity fibromuscular dysplasia. J Vasc Surg.

[5] Kadoya Y, Zen K, Matoba S (2017). Intraluminal fibrous webs in brachial artery fibromuscular dysplasia. JACC Cardiovasc Interv.

[6] Suzuki H, Daida H, Sakurai H, Yamaguchi H (1999). Familial fibromuscular dysplasia of bilateral brachial arteries. Heart.

[7] Alanore L, Perdu J, Plouin PF (2007). Dysplasie fibromusculaire artérielle. Presse Med.

[8] Verdure P, Triquenot-Bagan A, Perdu J (2008). Dissections artérielles cervicales multiples chez deux frères: dysplasie fibro-musculaire ou maladie du tissu conjonctif?. Rev Neurol (Paris).

[9] Cutts S, Grewal RS, Downing R (2000). Bilateral brachial artery fibromuscular dysplasia. Eur J Vasc Endovasc Surg.

[10] Rice RD, Armstrong PJ (2010). Brachial artery fibromuscular dysplasia. Ann Vasc Surg.

