# Supplementary material for: Fibromuscular dysplasia of the brachial artery diagnosis by Doppler ultrasound: a case report
Source: J Vasc Bras. 2026 Jan 12;24:e20250014. doi: 10.1590/1677-5449.202500142 (PMC12822372; doi:10.1590/1677-5449.202500142)
Supplement: Translation

# Translation: Displasia fibromuscular de artéria braquial diagnosticada por ultrassom Doppler: um relato de caso

**DOI:** 10.1590/1677-5449.202500141

**Authors:** Mariana Jordão França, Luciana Akemi Takahashi, Graciliano José França

**Affiliations:** 1 Universidade Positivo – UP, Curitiba, PR, Brasil.; 2 Universidade Federal do Paraná – UFPR, Curitiba, PR, Brasil.; 3 Pontifícia Universidade Católica do Paraná – PUCPR, Curitiba, PR, Brasil.

**Keywords:** displasia fibromuscular, artéria braquial, ultrassom Doppler

## Abstract

A displasia fibromuscular é uma doença vascular não inflamatória que acomete artérias de médio calibre e cuja etiologia permanece desconhecida. A doença predomina em mulheres caucasianas de meia-idade, afetando majoritariamente a artéria renal. O envolvimento das artérias dos membros superiores e inferiores é raro. Relatamos um caso raro de displasia fibromuscular da camada média da artéria braquial em uma paciente assintomática, identificada por meio de ultrassom Doppler.

## INTRODUÇÃO

A displasia fibromuscular (DFM) foi descrita pela primeira vez por Leadbetter & Burkland, em 1938, como uma doença vascular não inflamatória e não aterosclerótica, caracterizada pelo estreitamento de artérias de médio calibre decorrente de alterações fibrodisplásicas nas camadas arteriais^1^. Sua classificação baseia-se na camada primariamente acometida: íntima, média ou adventícia. A lesão da camada média ocorre em 90% dos casos e apresenta progressão lenta. O envolvimento dessa camada é reconhecido como o padrão clássico de imagem de colar de pérolas, em que há o espessamento e afinamento alternados dos segmentos da artéria acometida pela DFM. O acometimento da camada íntima manifesta-se como estenose focal e concêntrica, enquanto, na camada adventícia, apresenta-se sob a forma de estenose tubular. Essa doença pode também provocar ruptura da lâmina elástica interna, associada à presença um aneurisma, ou levar ao espessamento por lesões displásicas, resultando em estenose^2^. A presença de ao menos uma lesão arterial focal ou multifocal é necessária para estabelecer o diagnóstico de DFM. Já a presença isolada de aneurisma, dissecção ou tortuosidade é insuficiente para estabelecer esse diagnóstico^3^.

A DFM afeta predominantemente mulheres caucasianas, magras, com idade entre 15 e 50 anos^2^. Aproximadamente 80-90% dos casos ocorrem em mulheres^3^, e sua etiologia permanece desconhecida. As artérias mais comumente acometidas pela DFM são a renal e a carótida^4^. Estima-se que a prevalência dessa condição seja inferior a 1% da população, sendo que 60-75% dos casos envolvem as artérias renais. Além disso, foi descrito o acometimento de artérias viscerais, ilíacas, subclávias, poplíteas e braquiais^2^. O envolvimento das artérias dos membros superiores na DFM é considerado raro^4^. O padrão-ouro para avaliação por imagem é a angiografia por cateter^5^, embora possam ser utilizados outros métodos, como angiotomografia computorizada, angiorressonância magnética e ultrassom Doppler^2^. Relatamos o caso de uma paciente do sexo feminino com DFM unilateral da artéria braquial, assintomática, diagnosticada por meio de ultrassom Doppler.

O estudo foi aprovado pelo Comitê de Ética de nossa instituição (parecer nº 6.860.303). O termo de consentimento livre e esclarecido foi obtido para todos os procedimentos envolvendo seres humanos. A paciente assinou o termo de consentimento específico para a divulgação das imagens ultrassonográficas e para a publicação do caso.

## DESCRIÇÃO DO CASO

Paciente do sexo feminino, 64 anos, com histórico de cefaleia associada à suspeita de arterite temporal, a qual foi tratada com prednisona 60 mg/dia, resultando em melhora dos sintomas. No entanto, não foi realizado diagnóstico confirmatório por meio de biópsia de artéria temporal. No exame físico, a paciente apresentava pulsos distais diminuídos no membro superior direito. Foi encaminhada pelo Serviço de Reumatologia à equipe de Cirurgia Vascular para realização de ultrassom Doppler arterial do membro superior, com o objetivo de investigar a diminuição do pulso. A paciente negou claudicação nos membros superiores.

O ultrassom Doppler arterial revelou múltiplas pequenas dilatações na artéria braquial direita, sem presença de ateromas (Figura 1), intercaladas com estenoses hemodinamicamente significativas, resultando em *aliasing* no modo colorido e aumento da velocidade de pico sistólico no modo Doppler (Figura 2). Esses achados são sugestivos do padrão de “colar de pérolas”, compatível com o diagnóstico de DFM. As artérias subclávia e axilar do membro superior direito apresentavam ondas de morfologia normal (Figura 3). As artérias radial e ulnar do mesmo membro não apresentavam estenoses locais; entretanto, a fluxometria Doppler revelou sinais de hipofluxo, confirmando o diagnóstico de estenoses hemodinamicamente significativas na artéria braquial (Figura 4) . Não havia evidência de DFM na artéria braquial esquerda. As artérias subclávia, axilar, braquial, radial e ulnar esquerdas estavam pérvias, com paredes regulares, sem ateromas e com fluxo dentro da normalidade, sem sinal de estenose. Não foi realizado exame complementar de ultrassom Doppler para avaliação das artérias carótidas. A paciente permanece assintomática e em seguimento clínico.

**Figura 1:** Power Doppler da artéria braquial direita demonstrando pequenas estenoses intercaladas com dilatações no padrão de “colar de pérolas”. As setas grossas indicam áreas de dilatação, enquanto as setas finas indicam áreas de estenose.

**Figura 2:** Artéria braquial apresentando áreas de *aliasing* no modo colorido, aumento da velocidade de pico sistólico e diminuição do índice de resistência arterial, compatíveis com estenoses hemodinamicamente significativas e sequenciais.

**Figura 3:** Ultrassom Doppler da artéria axilar direita, apresentando curvas de morfologia normal e sem sinais de estenose.

**Figura 4:** Ultrassom Doppler da artéria radial direita, evidenciando sinais de hipofluxo devido a estenose significativa proximal.

## DISCUSSÃO

A DFM ainda tem sua etiologia desconhecida, embora se acredite que exista um componente genético, devido ao maior acometimento em mulheres caucasianas, podendo estar associada ao antígeno de histocompatibilidade HLA-DRw6^2^. Suzuki et al. reportaram um caso familiar de DFM bilateral da artéria braquial envolvendo mãe e filha, o que reforça a hipótese do fator genético. Os autores também mencionam estudos que sugerem herança com padrão autossômico dominante e redução da penetrância no sexo masculino^6^. São necessários estudos adicionais para uma melhor compreensão do papel das variações genéticas na patogênese da DFM. Atualmente, não existe teste genético específico para essa doença, nem há recomendação de testagem genética para parentes assintomáticos de pacientes portadores de DFM^3^. O predomínio em mulheres e a descoberta de alguns casos durante a gestação também sugerem um possível papel do estrogênio na patogênese dessa doença vascular^7^.

Outros casos têm sido associados a tabagismo, distúrbios da coagulação, mutação do fator V de Leiden, presença de anticorpos antifosfolipídios e estresse mecânico^8^. Além disso, estresse físico e episódios de trauma também podem contribuir para o desenvolvimento da DFM, pois essas lesões provocam isquemia na parede arterial e comprometimento dos *vasa vasorum* ^9^.

O diagnóstico da doença no membro superior pode ser incidental em pacientes assintomáticos, ou realizado após suspeita clínica. A DFM nesse segmento envolve principalmente a artéria braquial, embora tenham sido relatados casos de acometimento das artérias axilar, subclávia, radial e ulnar. O padrão de envolvimento mais comum é multifocal, com a maioria dos pacientes assintomáticos, e frequentemente apresenta envolvimento bilateral^3^. As manifestações de DFM no membro superior incluem cianose, fenômeno de Raynauld, parestesia, fraqueza, presença de massa pulsátil, úlcera ou, em casos mais graves, gangrena distal dos dedos associada a microembolismo^4^. Esses sintomas podem decorrer de alterações no fluxo arterial ou da compressão de estruturas nervosas^1^. No exame físico, podem ser identificadas diminuição do pulso periférico, discrepância da pressão arterial entre os membros superiores e presença de sopro arterial^3^. É importante diferenciar a DFM da aterosclerose, uma vez que a DFM pode apresentar sintomas semelhantes aos da aterosclerose em pacientes com queixa de claudicação intermitente^2^.

Devido ao caráter progressivo da DFM, é necessário o acompanhamento periódico desses pacientes. O tratamento é recomendado apenas para indivíduos sintomáticos^2^. Um estudo prospectivo realizado por Nguyen et al., envolvendo 22 mulheres com DFM no membro superior, revelou que 51,7% delas eram assintomáticas^4^.

Diversas intervenções podem ser realizadas em pacientes sintomáticos, desde terapias medicamentosas até procedimentos invasivos. Entre os fármacos utilizados estão antiagregantes plaquetários, anticoagulantes e bloqueadores do canal de cálcio^4^. O primeiro consenso internacional sobre o diagnóstico e manejo da DFM recomenda que, na ausência de contraindicações à terapia antiplaquetária, seja administrada aspirina na dose diária de 75-100 mg a pacientes portadores de DFM, com o objetivo de prevenir complicações trombóticas e tromboembólicas^3^. Já os procedimentos invasivos indicados para pacientes sintomáticos incluem angioplastia percutânea transluminal, trombólise dirigida por cateter, simpatectomia e *bypass* cirúrgico^4^.

Como o diagnóstico e o manejo da DFM em membros superiores se baseiam principalmente em relatos de caso ou séries com poucos participantes, não há consenso quanto ao tratamento^4^. Intervenções endovasculares tendem a ser preferíveis em segmentos curtos, enquanto a cirurgia aberta é indicada para segmentos de grande extensão^10^.

Um estudo prospectivo conduzido por Nguyen et al. concluiu que a terapia medicamentosa isolada apresenta eficácia limitada no alívio completo dos sintomas. Nesses casos, a intervenção invasiva inicial pode ser a angioplastia primária. Em situações mais graves, pode ser necessária mais de uma intervenção, optando-se pelo *bypass* cirúrgico ou, em alguns casos, pela simpatectomia^4^.

O ultrassom Doppler, por ser um método de avaliação rápido, de baixo custo e amplamente disponível, constitui uma opção de exame de imagem para a investigação da DFM. Além disso, permite diferenciar a DFM da aterosclerose e investigar se o acometimento é bilateral ou unilateral.

## Data Availability

All data generated or analyzed, and Doppler ultrasound images, are included in this article and/or in the supplementary material.
